# X4 Tropic Multi-Drug Resistant Quasi-Species Detected at the Time of Primary HIV-1 Infection Remain Exclusive or at Least Dominant Far from PHI

**DOI:** 10.1371/journal.pone.0023301

**Published:** 2011-08-24

**Authors:** Jade Ghosn, Julie Galimand, Stéphanie Raymond, Laurence Meyer, Christiane Deveau, Cécile Goujard, Jacques Izopet, Christine Rouzioux, Marie-Laure Chaix, for the ANRS CO 06 PRIMO cohort

**Affiliations:** 1 Université Paris Descartes, EA 3620, CHU Necker-Enfants Malades, Paris, France; 2 Service de Médecine Interne et Maladies Infectieuses, AP-HP, CHU Bicêtre, Le Kremlin-Bicêtre, France; 3 Laboratoire de Virologie, AP-HP, CHU Necker-Enfants Malades, Paris, France; 4 Laboratoire de Virologie, INSERM U563, Hôpital Purpan, Toulouse, France; 5 Faculté de Médecine Paris-Sud, AP-HP, INSERM U1018, Université Paris-Sud, Hôpital Bicêtre, Service d'épidémiologie et de santé publique, Le Kremlin-Bicêtre, France; INSERM, France

## Abstract

Our objective was to analyze the evolution of resistance mutations (RM) and viral tropism of multi-drug-resistant (MDR) strains detected at primary HIV-1 infection (PHI). MDR HIV strain was defined as the presence of genotypic resistance to at least 1 antiretroviral of the 3 classes. Tropism determinations (CCR5 or CXCR4) were performed on baseline plasma HIV-RNA and/or PBMC-HIV-DNA samples, then during follow-up using population-based sequencing of V3 loop and phenotypic tests. Clonal analysis was performed at baseline for env, RT and protease genes, and for HIV-DNA env gene during follow-up. Five patients were eligible. At baseline, RT, protease and env clones from HIV-RNA and HIV-DNA were highly homogenous for each patient; genotypic tropism was R5 in 3 (A,B,C) and X4 in 2 patients (D,E). MDR strains persisted in HIV-DNA throughout follow-up in all patients. For patient A, tropism remained R5 with concordance between phenotypic and genotypic tests. Clonal analysis on Month (M) 78 HIV-DNA evidenced exclusively R5 (21/21) variants. In patient B, clonal analysis at M36 showed exclusively R5 variants (19/19) using both genotypic and phenotypic tests. In patient C, baseline tropism was R5 by genotypic test and R5/X4 by phenotypic test. An expansion of these X4 clones was evidenced by clonal analysis on M72 HIV-DNA (12/14 X4 and 2/14 R5 variants). In patient D, baseline tropism was X4 with concordance between both techniques and HIV-RNA and HIV-DNA remained X4-tropic up to M72, confirmed by the clonal analysis. Patient E harboured highly homogenous X4-using population at baseline; tropism was unchanged at M1 and M18. In all patients, the initial MDR population was highly homogenous initially, supporting the early expansion of a monoclonal population and its long-term persistence. X4-tropic variants present at baseline were still exclusive (patients D and E) or dominant (at least one time point, patient C) far from PHI.

## Introduction

Sexual transmission of HIV-1 resistant strains has been well documented [1]. The frequency of strains harbouring resistance to at least one antiretroviral drug at the time of primary HIV-1 infection (PHI) is stable in Europe over the last decade and reaches approximately 10–12% [2]–[5]. Moreover, despite a theoretically impaired fitness [6], multi-drug resistant (MDR) viral strains can also be transmitted via the sexual route and establish themselves as the dominant viral population by massively fuelling the cellular reservoir [7]. Thus, unlike HIV-1 strains developing resistance mutations on a failing therapy during chronic disease, resistant HIV-1 strains identified at the time of PHI persist in plasma over time in a drug-free environment [7], [8]. In addition, transmission of X4-tropic HIV-1 strains at the time of PHI has also been documented, and the prevalence of X4 strains at the time of PHI reaches approximately 15% [9]–[11]. Long-term evolution of such X4 strains present at the time of PHI is unknown. The clinical implications are of serious concern since multidrug resistance as well as X4-usage can result in treatment failure and rapid clinical progression [12]–[17]. Our objective was to characterize intracellular HIV-DNA in patients with a MDR HIV-1 strain detected at the time of PHI and to analyze the viral tropism in such patients. We analysed the temporal evolution of resistance patterns and viral tropism in plasma virions and in intracellular HIV-DNA extracted from peripheral blood mononuclear cells (PBMC). Moreover, to analyze extensively the viral tropism, we performed a clonal analysis at baseline and during the follow-up and we classified the virus as X4 or R5 using both genotypic and phenotypic methods.

## Results

### Baseline characteristics of patients

Between 1996 and December 2009, 968 patients were included in the ANRS PRIMO cohort. Among them, five patients (four men who have sex with men (MSM), one woman) harbored a MDR strain and were included. This sub- study is exhaustive as all the patients infected with a MDR strain and included in the French PRIMO cohort were analyzed. Median time between estimated date of infection and enrolment in the cohort was 33 days (range 18–71). Their immuno-virological characteristics and resistance mutational pattern at baseline are summarised in Table S1. Resistance mutational pattern was identical between plasma HIV-RNA and intracellular HIV-DNA in all patients. All five patients harboured a subtype B strain.

When using the SVMgeno2pheno algorithm or the genotypic rule for tropism determination, three patients (A, B, C) harboured a CCR5-using strain and the remaining two (D and E) were infected with a CXCR4-using virus at the time of PHI. Genotypic analysis yielded similar tropism results between HIV-RNA and HIV-DNA at baseline. These results were concordant when tropism determination was performed using the phenotypic assay on PBMCs, except for patient B who harboured a R5/X4 strain at baseline. None of the three patients harbouring an HIV-1 strain using CXCR4 co-receptor at the time of PHI was homozygous for deletion Delta32 in CCR5 gene (two patients were wild-type CCR5 homozygous and one was Delta32 heterozygous).

**Figure 1:**
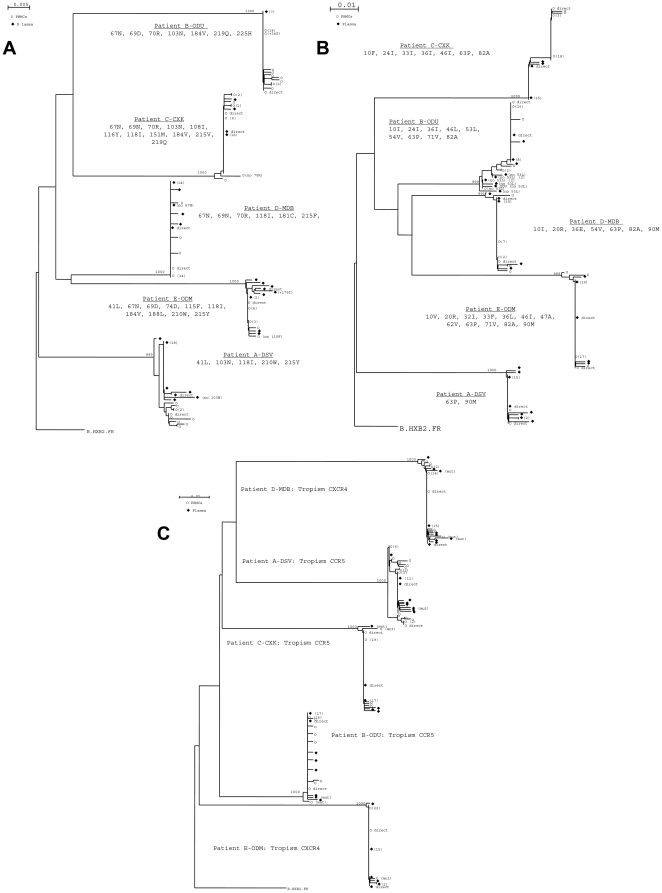
Phylogenetic analysis, based on the neighbour-joining method, of clones of HIV-1 reverse transcriptase gene sequences (1A), protease gene sequences (1B) and envelope gene sequences (1C) from patients A–E at baseline, demonstrating highly homogenous circulating and archived variants in each patient. Clones obtained from plasma HIV-1 RNA are in full squares and clones obtained from PBMC-HIV-1 DNA are in white circles. Resistance mutational pattern in reverse transcriptase gene and in protease gene is shown for each patient. Clones not harbouring all resistance mutations are indicated. HIV-1 tropism on Figure 1C is determined by genotypic test. Only bootstrap values ≥700 are shown. The reference sequence HXB2 was used as an outgroup. Genetic distance is indicated at the top of the figure, and represents the number of nucleotide substitutions per site. The numbers between brackets indicate the number of strictly identical sequences that segregate on the same branch.

For all five patients, clonal analysis based on reverse transcriptase (total 148 clones), protease (total 179 clones) and envelope (total 218 clones) genes from plasma HIV-RNA and PBMC-HIV-DNA showed a highly homogenous viral population (Figures 1A, 1B, 1C). Phylogenetic analysis showed intermingled sequences obtained from circulating virions and intra-cellular HIV-DNA. All mutations associated with NRTI and NNRTI resistance were all linked on the same genome in 144/148 variants and mutations associated with PI resistance were harboured on the same genome in four patients, patient E harbouring 10/40 clones without mutation F53L (Figure 1B).

At baseline, all variants present in plasma and in PBMC used the same co-receptor for a given patient. Clonal analysis of C2V3 region of *env* gene showed extremely homogenous viral population in each patient (A: 18/18 CCR5-using variants in HIV-RNA and 23/23 CCR5-using in HIV-DNA; B: 23/23 CCR5-using variants in HIV-RNA and 23/23 CCR5-using in HIV-DNA; C: 21/21 CCR5-using variants in HIV-RNA and 23/23 CCR5-using in HIV-DNA; D: 23/23 CXCR4-using variants in HIV-RNA and 21/21 CXCR4-using in HIV-DNA; E: 20/20 CXCR4-using variants in HIV-RNA and 23/23 CXCR4-using in HIV-DNA) (Figure 1C and Table S1).

Based on pairwise evolutionary distances, the intra-individual variability ranged between 0.00% and 0.47% for reverse transcriptase sequences, 0.00% and 0.75% for protease sequences and 0.0031% and 0.94% for envelope sequences.

### Longitudinal assessment of genotypic resistance tests in plasma HIV-RNA and cell-associated HIV-DNA

Median follow-up since enrolment was 78 months (range 18–96). Results are summarised in Table S1. All resistance mutations persisted in plasma HIV-RNA in patient A up to Month 36 (M36) except for mutation K103N and V118I in reverse transcriptase gene that reverted to wild-type. This patient was successfully treated with persistence of resistant mutations in PBMC-HIV-DNA up to the end of follow-up (M78).

Resistance mutational pattern was unchanged in patient B in plasma HIV-RNA and HIV-DNA up to the end of follow-up (M36) except for mutation 184V that reverted to wild-type M184 in plasma HIV-RNA sampled at M12; this mutation persisted however in HIV-DNA throughout the 36-month follow-up. Reverse transcriptase mutations were unchanged in patient C up to M24 (except for mutation V118I), genotypic resistance test could not be performed thereafter. Patient D started HAART early after PHI, resistance mutational pattern was unchanged (except for mutation K70R which reverted to wild-type and mutation T215F which shifted to T215S) in HIV-DNA up to M72, and amplification of HIV-DNA failed on the last sample available (M84) probably due to very low HIV-DNA load. Patient E was rapidly treated for hepatitis C infection with pegylated interferon α and ribavirine, both HIV-RNA and HIV-DNA fell to undetectable levels as soon as M18.

### Longitudinal assessment of viral tropism of HIV-DNA in PBMC

**Figure 2:**
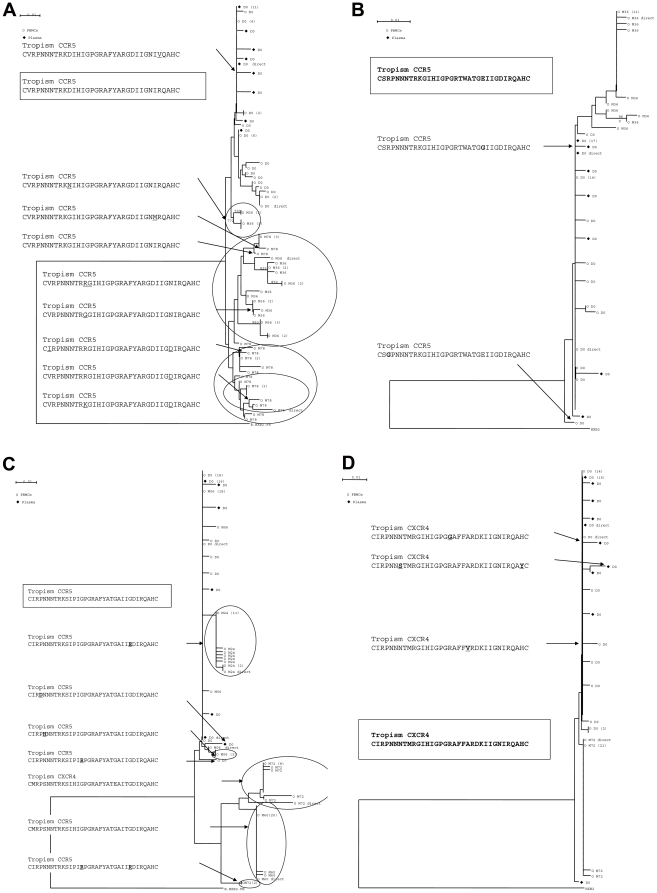
Longitudinal phylogenetic analysis, based on the neighbour-joining method, of clones of HIV-1 envelope gene from patient A (2A), B (2B), C (2C) and E (2D). Clones obtained from plasma HIV-1 RNA are in full squares and clones obtained from PBMC-HIV-1 DNA are in white circles. The most frequent amino-acid sequence of the V3-loop is indicated for each patient in a square. Amino-acid substitutions within the V3-loop are underlined and correspondent variants are indicated by an arrow for each patient. Only bootstrap values ≥700 are shown. The reference sequence HXB2 was used as an outgroup. Genetic distance is indicated at the top of the figure, and represents the number of nucleotide substitutions per site. The numbers between brackets indicate the number of strictly identical sequences that segregate on the same branch.

Results of tropism determination with SVMgeno2pheno algorithm, the genotypic rule and the phenotypic assay are summarised in Table S1. For patient A, harbouring a CCR5 strain at baseline, viral tropism determined by whole population sequencing was unchanged in PBMC at M36 and M78. Results were strictly concordant between genotypic and phenotypic tests at baseline and M78. Clonal analysis of C2V3 region of *env* gene from HIV-DNA extracted from available PBMC samples showed exclusively homogenous variants, 22/22 CCR5-using variants at M36 and 21/21 CCR5 at M78 (Figure 2A).

Patient B was infected with a highly homogenous CCR5-using population. The follow-up of patient B revealed a CCR5 strain determined by genotypic methods at M12, M24 and M36. Results were strictly concordant between genotypic and phenotypic tests, at M36. Clonal analysis revealed 19/19 CCR5-using variants at M36 on PBMCs (Figure 2B).

Patient C was infected with a highly homogenous CCR5-using population, determined by genotypic method, at the time of PHI. Interestingly, the phenotype was R5/X4 at baseline while the genotype was CCR5 both with the SVMgeno2pheno algorithm and the genotypic rule. During follow-up, viral tropism determined by whole population sequencing showed a CCR5 virus at M6, M24, M60. Clonal analysis of C2V3 region from PBMC-HIV-DNA showed exclusively CCR5-using variants at M6 (23 clones), M24 (18 clones), M60 (22 clones). We observed a change in the viral tropism in blood cells at M72 with a CXCR4 virus determined by both genotypic techniques. At the end of the follow-up (M96), viral tropism determined by bulk-sequencing was still CCR5 while it was CXCR4 at M72. This result was confirmed by the clonal analysis which revealed 12/14 clones CXCR4 and 2/14 clones CCR5 (Figure 2C).

Patient D was infected with a highly homogenous CXCR4-using population determined by both genotypic and phenotypic methods. Viral tropism determined by whole population sequencing was unchanged in PBMC at M48, M72 and M84. Phenotypic test was unavailable at M84. Clonal analysis showed exclusively homogenous variants, 23/23 CXCR4-using variants at M72 (Figure 2D).

Patient E was infected with a highly homogenous CXCR4-using population. Viral tropism was unchanged at M1 in plasma (determined by both genotypic and phenotypic methods) and at M18 determined by whole population sequencing on blood cells.

## Discussion

This is the first study providing longitudinal data on both resistance mutational pattern and viral tropism of circulating and archived viral strains in patients infected with a MDR HIV-1 as soon as primary HIV infection. This is an exhaustive study as all the patients infected with a MDR strain and included in the ANRS PRIMO cohort were analyzed in this sub-study. We confirm that HIV-1 MDR strains can be transmitted via the sexual route; they massively fuel the cellular reservoir at the earliest time point after infection and establish themselves as the dominant viral population [7]. Indeed, these five patients acquired HIV-1 through sexual intercourse and no other risk factor for HIV-1 acquisition was declared. Resistance mutations in reverse transcriptase and in protease genes were evident in both circulating virions and intra-cellular HIV-DNA after a median of 33 days after estimated date of infection. Moreover, clonal analysis of the reverse transcriptase and protease genes from plasma HIV-RNA and from HIV-DNA showed a highly homogenous viral population in all 5 patients, with no specific compartmentalization in the cellular compartment. Resistant strains persisted in plasma HIV-RNA and in infected blood cells in a drug-free environment, but they also persisted in the cellular reservoir in patients with suppressed viral replication on HAART. Despite the usual dynamic processes affecting the pool of infected cells [18], HIV-DNA isolated from circulating PBMC far from PHI still exhibited the same resistance mutational pattern than at the time of PHI, indicating that resistant viruses entering the cellular reservoir early in infection had not been replaced. This is supported by the detection of a highly homogenous and exclusively resistant viral population in the cellular reservoir at the earliest time-point after infection, with no wild-type variants to overgrow the resistant and probably less fit variants [19]. Interestingly, mutation M184V with deleterious impact on viral fitness [20], [21] shifted rapidly to wild-type amino-acid residue in plasma for patient E in the absence of drug-selective pressure. Reversion of 184V acquired at the time of PHI has already been described in plasma HIV-RNA [22], [23]. However, even if no longer detectable in plasma, we showed that this resistance mutation persisted in the cellular reservoir up to M36, which makes rapid re-emergence possible in plasma if lamivudine or emtricitabine were to be started. Other mutations with low impact on replicative capacity shifted also to wild-type amino-acid residue during follow-up and in the absence of drug-selective pressure.

HIV-1 binds the CCR5 co-receptor early in the course of HIV infection, and that X4-using viruses emerge later in the course of HIV disease [24]–[26]. Balandya et al recently showed that semen promotes the preferential transmission of R5 tropic HIV strains [27]. Here we show that CXCR4-using viruses can be sexually transmitted. Indeed, monophyletic exclusively CXCR4-using variants were detected in plasma and archived in PBMC of two patients (D and E) soon after infection with both genotypic and phenotypic techniques (SVMgeno2pheno algorithm and genotypic rule; and phenotypic assay). For patient C, we observed a discrepancy between the genotypic algorithm/rule which classified the virus as R5 and the phenotypic assay which identified a X4 virus. Of note, none of these three patients (C, D, E) was homozygous for deletion Delta32 in CCR5 gene. This discordance between phenotypic and genotypic methods in patient C could be explained by a better sensitivity of the phenotypic assay in detecting minor amounts of CXCR4-using viruses [28]. Moreover during the follow-up of this patient, the clonal analysis at M72 revealed a mixture of CXCR4-using clones and CCR5-using clones. One limitation with our cloning method is the fact that any similar sequences coming from a single PCR reaction tube are likely to represent PCR resampling. The single genome amplification would have offered a more reliable approach, but this method is sometimes difficult to perform with limited material to start with. Interestingly, Jordan et al have recently showed that either method is likely to provide a similar measure of population diversity, provided that an adequate number of PCR templates is analyzed [29]. Here we analyzed a high number of PCR templates at baseline, and we are conscious that our method might not be representative of all variants during follow-up in treated patients with low plasma HIV-RNA and low intracellular HIV-DNA.

Recent reports suggest that 6–17% of patients get infected with an CXCR4-using virus at the time of PHI [9]–[12], [17]. This finding is clinically relevant since X4-usage as soon as PHI has been associated with severe and rapid progression of HIV disease [12], [13]. Indeed, CD4 cell count was low in patients D and E as soon as the diagnosis of PHI. Of note, patients C, D and E who harboured CXCR4-using variants at the time of PHI were also infected with a MDR strain. In a previous study included 390 patients at the time of PHI, no association was found between the presence of resistant virus and HIV tropism [9]. However, a trend towards lower CD4 cell count was observed in patients infected with a resistant X4/DM-tropic virus in comparison with patients infected with a resistant CCR5-tropic strain. Such patients might be at high risk of rapid disease progression [15] and most importantly, they have limited therapeutic options as soon as PHI. Moreover, they might be at high risk of fast decline in CD4 cell count as it was reported in 78 subjects who completed a 12 month follow-up without undergoing antiretroviral therapy [11]. Other studies demonstrated an increased risk of developing a clinical event among patients infected with X4 tropic viruses compared with R5 viruses [30]. In the MACS cohort, HIV-1 CXCR4-using was detected more frequently among men who developed AIDS≤11 years after seroconversion than among those who did not [31]. In the Swiss HIV Cohort Study, the authors showed that HIV-1 coreceptor usage and CXCR4-specific viral loads strongly predicted disease progression during cART, independent of CD4 cell count and total viral load [32]. In our study, one patient (E) described herein and harbouring an CXCR4-using virus at the time of PHI was not started on HAART until M18, with no arguments for rapid disease progression or CD4 decline. The two other patients (patient C and patient D) harbouring an CXCR4-using strain at the time of PHI were started on HAART at the time of PHI until M38 and M30 respectively. After HAART interruption, the two patients were followed until M96 and M84 without disease progression or CD4 decline. Of note, the presence of multiple resistance mutations in key genes (reverse transcriptase and protease) might affect viral replicative capacity, thus rendering such a viral variant less aggressive even if it uses the CXCR4 co-receptor. However, Markowitz et al have reported on a patient infected with a MDR and CXCR4-using strain, who experienced rapid CD4 decline and progression of HIV-related disease soon after PHI [15]. Of note, as from M6 after PHI, plasma HIV-RNA was somehow low in our patients when untreated, suggesting they might be HIV-controllers. Less than 4% of patients enrolled in the ANRS PRIMO cohort exhibited such a spontaneous control of HIV-1 replication [33].

In chronically HIV-1 infected antiretroviral naïve patients starting HAART, viral tropism was not modified within one year after HAART initiation [34]. The temporal evolution of CXCR4-using viruses acquired at the time of PHI has been poorly documented. We determined HIV-1 tropism on cell-associated viruses using HIV-DNA extracted from PBMC on the samples available during the follow-up. In contrast, most studies have carried out tropism tests on HIV-RNA extracted from the plasma. A recent report by Raymond et al described the good agreement between HIV-1 tropism in PBMC and in plasma at the stage of PHI [12]. Here, we show that the viral population remained exclusively R5 (patients A and B) or X4 (patients D and E) far from PHI, supporting the early expansion of a monoclonal viral population at the time of primary infection. In contrast, a mixture of viruses was evident in patient C far from PHI. At baseline, the original virus remained R5 with the genotypic tools while it was found X4/R5 with the phenotypic assay. At M72, clonal analysis evidenced a mixture of R5 and CXCR4-tropic variants. There are four hypotheses to explain this finding. First, variants identified by our cloning method might not be representative of all variants present in the cellular reservoir at the time of PHI; we thus might have missed some CXCR4-using variants in patient C. Of note, the sensitivity of the TTT assay for detecting minor amounts of CXCR4-using viruses was 0.5% [28]. Second, immune pressure exerted on *env* gene might have promoted genetic evolution within the V3 loop. This hypothesis is supported by the fact that, as soon as PHI, intra-individual variability was highest in *env* gene than in *reverse transcriptase* or *protease* genes. Third, the evolution of co-receptor usage in some variants might have been promoted by down or up regulations of the expression of these co-receptors on the surface of target cells [35]. The fourth hypothesis would be a recirculation of CXCR4-using strains archived at PHI in the cellular reservoir and which persisted far from PHI. Overall, our data suggest that CXCR4-tropic variants present at the time of PHI remain exclusive or at least dominant far from PHI, with no possible use of CCR5-antagonists in these patients even far from PHI.

Despite the usual dynamic process affecting the pool of infected cells, MDR and CXCR4-using HIV-strains archived at PHI in the cellular reservoir persisted far from PHI throughout a median 78-month follow-up. Overall, these data suggest that determination of viral tropism at the time of PHI might be useful in order to closely monitor patients harbouring a CXCR4-using strain. Further studies are needed to assess whether the evidence of a CXCR4-using virus during PHI can be used as a diagnostic tool with clinical relevance rather than a prognostic marker.

## Methods

### Study population

Our study comprised patients presenting with PHI, enrolled in the multicentre prospective French ANRS PRIMO Cohort (ANRS CO6) [36]–[38]. The ethics committee of Cochin Hospital approved the study and all patients gave their written informed consent. This observational cohort does not impose guidelines for systematic treatment of patients presenting with PHI, the decision of initiating highly active antiretroviral therapy (HAART) or not relying on the primary care physician in each clinical setting on the basis of the French guidelines [39].

Primary HIV-infection was identified as previously described [40]. Briefly, primary HIV-infection had to be assessed by: (a) a negative or indeterminate HIV ELISA associated with a positive antigenemia or plasma HIV RNA (b) a Western blot profile compatible with ongoing seroconversion (incomplete WB with absence of antibodies to pol proteins) or (c) an initially negative test for HIV antibody followed within 6 months by a positive HIV serology.

For all patients enrolled in the PRIMO Cohort, blood plasma and PBMC or whole blood were collected at inclusion before any treatment initiation. Patients enrolled in the present study were selected if (i) they harboured HIV-1 multi-drug-resistant (MDR) virus in plasma at inclusion defined as: resistant to at least one nucleoside analogue reverse transcriptase inhibitor (NRTI) and one non-nucleoside reverse transcriptase inhibitor (NNRTI) and one protease inhibitor (PI) at time of PHI, and (ii) who had a minimum of 18-month-follow-up with available stored blood plasma and peripheral blood mononuclear cells (PBMC) samples.

### Quantification of HIV-RNA in blood plasma

HIV RNA was quantified by using the Cobas taqMan HIV-1 v2.0 assay kit (Roche Diagnostics, Meylan, France) according to the manufacturer's instructions.

### Quantification of HIV-DNA in peripheral blood mononuclear cells

PBMC were isolated from fresh whole blood by centrifugation on a one-layer Ficoll Hypaque gradient. PBMC were washed three times in RPMI medium, then counted and kept as dry pellets at −80°C. Total DNA was extracted from 200 µl of whole blood or from PBMC pellet using QIAamp DNA mini kit (Qiagen, Courtaboeuf, France) and HIV-DNA quantified by real-time PCR (HIV-DNA Biocentric kit, Bandol, France). The real-time PCR targets a conserved consensus region in the long terminal repeat (LTR) region of the HIV-1 major group [41], [42]. Results were expressed as log_10_ number of HIV-DNA copies per 10^6^ PBMC.

### Genotypic resistance tests

For all patients enrolled in the PRIMO cohort, genotypic resistance tests were systematically performed. The HIV-1 reverse transcriptase (RT) and protease genes were amplified from plasma HIV-RNA and cell-associated HIV-DNA by a single RT-PCR followed by nested PCR using published primers [43]. For HIV-DNA, one microgram of DNA extracted from total PBMC was amplified by PCR. PCR final products were visualized on gels then purified using the QIAquick PCR Purification HIV kit (Qiagen,Courtaboeuf, France.). After purification, PCR products were sequenced using the fluorescent dideoxy-terminator method (Big Dye Terminator kit, Applied Biosystem, Perkin Elmer, Foster City, Calif.) on an ABI 3130 Genetic Analyser sequencer (Applied Biosystem). Sequences were aligned using Sequence Navigator® software. The amino acids at codons associated with resistance to NRTI, NNRTI and PI were identified according to the 2010 International AIDS Society list (www.iasusa.org). HIV drug resistance was defined according to the 2010 HIV-1 genotypic resistance interpretation algorithm of the French National Agency for Research on AIDS (ANRS) (www.hivfrenchresistance.org). In this study, genotypic resistance tests were performed on paired stored samples of plasma and PBMC or whole blood collected at baseline, at month 6 (M6), M12, M18, M24 and then every twelve months when available.

### Genotypic methods for determining virus tropism

The *env* (340 bp) C2V3 regions were amplified from plasma HIV-RNA and intra-cellular HIV-DNA as described elsewhere [9]. We determined the HIV-1 co-receptor usage of each sample by two genotypic methods. One was the SVMgeno2pheno algorithm (available at: http://coreceptor.bioinf.mpi-sb.mpg.de/cgi-bin/coreceptor.pl). We chose a false-positive rate of 5% for this test to obtain a high specificity (95%) for the detection of CXCR4 variants. The other was a genotypic rule based on amino acid residues at positions 11 and 25 and the overall net charge of V3 [44]–[46]. One of the following criteria was required for predicting X4 co-receptor usage: (i) R or K at position 11 of V3 and/or K at position 25, (ii) R at position 25 of V3 and a net charge of ≥+5, or (iii) a net charge of ≥+6 [28], [47], [48]. The V3 net charge was calculated by subtracting the number of negatively charged amino acids (D and E) from the number of positively charged ones (K and R). Viruses were classified into two categories: those with the absence (R5) or presence of X4-tropic viruses (X4 or X4/DM). As the genotypic tests are unable to predict if the viruses used only the CXCR4 or both the CCR5 and the CXCR4, we decided to call them X4 for more clarity of the paper.

HIV-1 co-receptor usage of plasma virions and cell associated virus was determined at baseline and on HIV-DNA extracted from PBMC samples available during follow-up.

### Phenotypic method for determining virus tropism

Phenotypic characterization of HIV-1 tropism was performed using the Toulouse Tropism Test (TTT) assay [49]. A region spanning gp120 and the ectodomain of the gp41 *env* gene of plasma HIV-RNA was amplified by reverse transcription PCR. The phenotype of HIV-1 coreceptor usage was determined using a recombinant virus entry assay, as previously described [28]. The sensitivity of the assay for detecting minor amounts of CXCR4-using viruses was 0.5% [28].

### Determination of the Delta32 deletion in CCR5 gene

Determination of the Delta32 deletion in CCR5 gene was performed in patients harbouring an HIV-1 strain using CXCR4 co-receptor at the time of PHI, as described previously [50].

### Clones of HIV-RNA and HIV-DNA of reverse transcriptase, protease and envelope genes

In order to characterize the variants present in plasma and in the cellular blood reservoir at baseline, i.e. early after PHI, HIV-DNA *reverse transcriptase*, *protease and envelope* genes PCR products from plasma and PBMC samples were cloned into the pGEM-T Easy Vector System (Promega, Charbonnières, France) and transformed into Escherichia coli JM109 competent cells (Promega) as recommended by the firm. After overnight incubation at 37°C, insertion was checked by PCR with inner primers on white colonies. Each PCR product with the correct molecular weight was purified with the QIAquick PCR purification kit (Qiagen) and then sequenced as described above. In addition, clones of *envelope* gene were performed on intracellular HIV-DNA extracted from the latest available PBMC sample collected during follow-up. GenBank accession numbers are JN181579-JN181727 and JN193569-JN194125.

### Phylogenetic analysis

All sequences of HIV-RNA and HIV-DNA reverse transcriptase, protease and envelope genes were aligned with Clustal X 2.0.11 ® software. Pairwise evolutionary distances were estimated with DNADist, using Kimura's two-parameter method, then the phylogenetic trees were constructed by a neighbour joining method (neighbour program implemented in the Phylip package) [51]. The reliability of each tree topology was estimated from 1000 bootstrap replicates [51].

## Supporting Information

Table S1 **Immunological,-virological characteristics and resistance mutation patterns at baseline and during the follow-up.** A: patient A-DSV, B: patient B-ODU, C: patient C-CXK, D: patient D-MDB, E: patient E-ODM. D: day, M: month, RT: reverse transcriptase, nd: not done, TTT: Toulouse Tropism Test. TDF: tenofovir, FTC: emtricitabine, ZDV: zidovudine, NFV: nelfinavir, EFV: efavirenz, FosAPV: fos-amprenavir. HIV-DNA: log_10_ copies/10^6^ PB.
(DOC)
