# Supplementary material for: X4 Tropic Multi-Drug Resistant Quasi-Species Detected at the Time of Primary HIV-1 Infection Remain Exclusive or at Least Dominant Far from PHI
Source: PLoS One. 2011 Aug 24;6(8):e23301. doi: 10.1371/journal.pone.0023301 (PMC3160852; doi:10.1371/journal.pone.0023301)
Supplement: Table S1 — Immunological,-virological characteristics and resistance mutation patterns at baseline and during the follow-up. A: patient A-DSV, B: patient B-ODU, C: patient C-CXK, D: patient D-MDB, E: patient E-ODM. D: day, M: month, RT: reverse transcriptase, nd: not done, TTT: Toulouse Tropism Test. TDF: tenofovir, FTC: emtricitabine, ZDV: zidovudine, NFV: nelfinavir, EFV: efavirenz, FosAPV: fos-amprenavir. HIV-DNA: log10 copies/106 PB. (DOC) [file pone.0023301.s001.doc]

|  | Treatment | Time | Sample | CD4  /mm^3^ | HIV-RNA log_10_ | RT resistance mutations | Protease resistance mutations | Phenotropism  (TTT) | Genotropism (Rule 11/25 + V3 net charge) | Genotropism  (Geno2pheno 5%) | Genotropism Clones (Geno2pheno 5%) |
| --- | --- | --- | --- | --- | --- | --- | --- | --- | --- | --- | --- |
| A | Untreated | D0 | Plasma | 605 | 2.69 | 41L, 103N, 118I, 210W, 215Y | 63P, 90M | *nd* | **R5** | **R5** | **18R5** |
|  |  |  | PBMC |  | 1.84 | 41L, 103N, 118I, 210W, 215Y | 63P, 90M | **R5** | **R5** | **R5** | **23R5** |
|  | TDF/FTC/FosAPV/r | M36 | Plasma | 302 | 2,88 | 41L, 184V, 210W, 215C | 63P, 90M | *nd* | **R5** | **R5** | *nd* |
|  |  |  | PBMC |  | *nd* | *nd* | *nd* | *nd* | **R5** | **R5** | **22R5** |
|  | TDF/FTC/FosAPV/r | M78 | Plasma | 465 | <1,70 | *nd* | *nd* | *nd* | *nd* | *nd* | *nd* |
|  |  |  | PBMC |  | *nd* | 41L, 118I, 210stop, 215C | 63P, 90M | **R5** | **R5** | **R5** | **21R5** |
| B | Untreated | D0 | Plasma | 725 | 2,95 | 67N, 69D, 70R, 103N, 184V, 219Q, 225H | 10I, 24I, 36I, 46L, 53L, 54V,  63P, 71V, 82A | *nd* | **R5** | **R5** | **23R5** |
|  |  |  | PBMC |  | 2,89 | 67N, 69D, 70R, 103N, 184V, 219Q, 225H | 10I, 24I, 36I, 46L, 53L, 54V,  63P, 71V, 82A | **R5** | **R5** | **R5** | **23R5** |
|  | Untreated | M12 | Plasma | 993 | 2.96 | 67N, 69D, 70R, 103N, 219Q, 225H | 10I, 24I, 36I, 46L, 53L, 54V, 63P, 71V, 82A | *nd* | **R5** | **R5** | *nd* |
|  |  |  | PBMC |  | *nd* | 67N, 69D, 70R, 103N, 184V, 219Q, 225H | 10I, 24I, 36I, 46L, 54V, 63P, 71V, 82A | *nd* | **R5** | **R5** | *nd* |
|  | Untreated | M24 | Plasma | 766 | 2.35 | 67N, 69D, 70R, 103N, 219Q, 225H | 10I, 24I, 36I, 46L, 54V, 63P, 71V, 82A | *nd* | **R5** | **R5** | *nd* |
|  |  |  | PBMC |  | *nd* | *nd* | 10I, 24I, 36I, 46L, 54V, 63P, 71V, 82A | *nd* | **R5** | **R5** | *nd* |
|  | Untreated | M36 | Plasma | 751 | 3,16 | 67N, 69D, 70KR, 103N, 219Q, 225H | 10I, 24I, 36I, 46L, 54V,  63P, 71V, 82A | *nd* | **R5** | **R5** | *nd* |
|  |  |  | PBMC |  | *nd* | 67N, 69D, 70R, 103N, 184V, 219Q, 225H | 10I, 24I, 46L, 54V,  63P, 71V, 82A | **R5** | **R5** | **R5** | **19R5** |
| C | ZDV+3TC+NFV | D0 | Plasma | 593 | 4.33 | 67N, 69N, 70R, 103N, 108I, 116Y, 118I, 151M, 184V, 215V, 219Q | 10F, 24I, 33I, 36I, 46I, 63P, 82A | *nd* | **R5** | **R5** | **21R5** |
|  |  |  | PBMC |  | 2.98 | 67N, 69N, 70R, 103N, 108I, 116Y,118I, 151M, 184V, 215V, 219Q | 10F, 24I, 33I, 36I, 46I, 63P, 82A | **R5/X4** | **R5** | **R5** | **23R5** |
|  | ZDV+3TC+NFV | M6 | Plasma | 860 | <1,70 | *nd* | *nd* | *nd* | *nd* | *nd* | *nd* |
|  |  |  | PBMC |  | *nd* | 67N, 69N, 70R, 103N, 108I, 116Y, 151M, 184V, 215V, 219Q | *nd* | **R5/X4** | **R5** | **R5** | **23R5** |
|  | ZDV+3TC+NFV | M24 | Plasma | 789 | <1,70 | *nd* | *nd* | *nd* | *nd* | *nd* | *nd* |
|  |  |  | PBMC |  | 2.00 | 67N, 69N, 70R, 103N, 108I, 116Y, 151M, 184V, 215V, 219Q | *nd* | *nd* | **R5** | **R5** | **18R5** |
|  | Untreated | M60 | Plasma | 866 | <1.70 | *nd* | *nd* | *nd* | *nd* | *nd* | *nd* |
|  |  |  | PBMC |  | 1.25 | *nd* | *nd* | *nd* | **R5** | **R5** | **22R5** |
|  | Untreated | M72 | Plasma | 569 | <1.70 | *nd* | *nd* | *nd* | *nd* | *nd* | *nd* |
|  |  |  | PBMC |  | *nd* | *nd* | *nd* | *nd* | **R5/X4** | **R5/X4** | **12X4/2R5** |
|  | Untreated | M96 | Plasma | 946 | <1.70 | *nd* | *nd* | *nd* | *nd* | *nd* | *nd* |
|  |  |  | PBMC |  | *nd* | *nd* | *nd* | *nd* | **R5** | **R5** | ND |
| D | ZDV+3TC+EFV | D0 | Plasma | 461 | 5.11 | 67N, 69N, 70R, 118I, 181C, 215F, 219Q | 10I, 20R, 36I, 54V, 63P,  71V, 82A, 90M | *nd* | **X4** | **X4** | **23X4** |
|  |  |  | PBMC |  | 3.33 | 67N, 69N, 70R, 118I, 181C, 215F, 219Q | 10I, 20R, 36I, 54V, 63P,  71V, 82A, 90M | **R5/X4** | **X4** | **X4** | **21X4** |
|  | Untreated since M30 | M48 | Plasma | 765 | <1,70 | *nd* | *nd* | *nd* | *nd* | *nd* | *nd* |
|  |  |  | PBMC |  | *nd* | *nd* | *nd* | *nd* | **X4** | **X4** | *nd* |
|  | Untreated | M72 | Plasma | 561 | 2.78 | 67N, 69D, 118I, 181C, 215S, 219Q | 10I, 20R, 36I, 54V, 63P, 71V, 82A, 90M | *nd* | *nd* | *nd* | *nd* |
|  |  |  | PBMC |  | *nd* | *nd* | *nd* | *nd* | **X4** | **X4** | **23X4** |
|  | Untreated | M84 | Plasma | 561 | 2,78 | *nd* | *nd* | *nd* | *nd* | *nd* | *nd* |
|  |  |  | PBMC |  | *nd* | *nd* | *nd* | *nd* | **X4** | **X4** | *nd* |
| E | Untreated for HIV  Treated with Peg-intreferon + Ribavirin for HCV | D0 | Plasma | 292 | 5,67 | 41L, 67N, 69D, 74V, 115F, 118I, 184V, 188L, 210W, 215Y | 10V, 20R, 32I, 33F, 36L, 46I, 47A, 62V, 63P, 71V, 82A, 90M | *nd* | **X4** | **X4** | **20X4** |
|  |  |  | PBMC |  | 2,97 | 41L, 67N, 69D, 74V, 115F, 118I, 184V, 188L, 210W, 215Y | 10V, 20R, 32I, 33F, 36L, 46I, 47A, 62V, 63P, 71V, 82A, 90M | **R5/X4** | **X4** | **X4** | **23X4** |
|  |  | M1 | Plasma | 337 | 2.65 | *nd* | *nd* | **R5X4** | **X4** | **X4** | *nd* |
|  |  | M18 | Plasma | 769 | <1,70 | *nd* | *nd* | *nd* | *nd* | *nd* | *nd* |
|  |  |  | PBMC |  | *nd* | *nd* | *nd* | *nd* | **X4** | **X4** | *nd* |

Table S1 : Immunological,-virological characteristics and resistance mutation patterns at baseline and during the follow-up

A : patient A-DSV, B : patient B-ODU, C : patient C-CXK, D : patient D-MDB, E : patient E-ODM

D : day, M : month, RT : reverse transcriptase, nd : not done, TTT : Toulouse Tropism Test

TDF : tenofovir, FTC : emtricitabine, ZDV : zidovudine, NFV : nelfinavir, EFV : efavirenz, FosAPV : fos-amprenavir.

HIV-DNA : log_10_ copies/10^6^ PBMC
